# Lung Disease Detection Using U-Net Feature Extractor Cascaded by Graph Convolutional Network

**DOI:** 10.3390/diagnostics14121313

**Published:** 2024-06-20

**Authors:** Pshtiwan Qader Rashid, İlker Türker

**Affiliations:** Department of Computer Engineering, Karabuk University, 78050 Karabuk, Turkey; pshtiwan572@gmail.com

**Keywords:** lung disease detection, graph convolutional networks, COVID-19, graph representative learning, deep learning

## Abstract

Computed tomography (CT) scans have recently emerged as a major technique for the fast diagnosis of lung diseases via image classification techniques. In this study, we propose a method for the diagnosis of COVID-19 disease with improved accuracy by utilizing graph convolutional networks (GCN) at various layer formations and kernel sizes to extract features from CT scan images. We apply a U-Net model to aid in segmentation and feature extraction. In contrast with previous research retrieving deep features from convolutional filters and pooling layers, which fail to fully consider the spatial connectivity of the nodes, we employ GCNs for classification and prediction to capture spatial connectivity patterns, which provides a significant association benefit. We handle the extracted deep features to form an adjacency matrix that contains a graph structure and pass it to a GCN along with the original image graph and the largest kernel graph. We combine these graphs to form one block of the graph input and then pass it through a GCN with an additional dropout layer to avoid overfitting. Our findings show that the suggested framework, called the feature-extracted graph convolutional network (FGCN), performs better in identifying lung diseases compared to recently proposed deep learning architectures that are not based on graph representations. The proposed model also outperforms a variety of transfer learning models commonly used for medical diagnosis tasks, highlighting the abstraction potential of the graph representation over traditional methods.

## 1. Introduction

Lung diseases are assumed to be a major global health concern, affecting millions of lives and placing a significant burden on healthcare systems worldwide [1]. Coronavirus disease (COVID-19), an infectious disease caused by the SARS-CoV-2 virus, significantly impacts human health, particularly those with lung disease, causing respiratory symptoms, pneumonia, acute respiratory distress syndrome (ARDS), and other conditions [2]. The early detection and accurate diagnosis of lung diseases are critical for effective treatment and improved patient health outcomes. Traditional diagnostic methods often rely on the human interpretation of medical images, leading to time-consuming and subjective assessments. However, the intersection of cutting-edge technology and medical science is opening new avenues to increase the precision and effectiveness of the early diagnosis of respiratory diseases [3].

Baratella et al. performed a study to compare how well traditional chest X-rays (CXR) and digital tomosynthesis (DTS) could find changes in the pulmonary interstitial spaces in people who had recovered from severe COVID-19. Two radiologists, each with 16 and 10 years of experience in chest imaging, independently assessed the DTS images to compare them with computed tomography (CT). The diagnostic accuracy of DTS was markedly superior to that of CXR in identifying interstitial lung abnormalities [4]. Bellini et al. conducted a study to assess the potential reduction in the lung volume in long-term COVID-19 patients with mild respiratory symptoms using the quantitative analysis of chest CT images. In patients with long COVID symptoms and no visible abnormalities on chest CT, quantitative volume analysis revealed an average reduction in the lung volume of 10% compared to individuals of the same age who had never had COVID-19. If a chest CT scan shows no signs of inflammation, doctors may mistakenly attribute moderate respiratory symptoms in young COVID-19 patients to anxiety. Upon examination, a chest CT scan revealed no evidence of inflammatory abnormalities [5]. Thoracic imaging is a multimodal technique that utilizes many types of waves to generate images of the organs located in the thorax. The medical imaging techniques used include chest X-rays, computed tomography (CT), lung ultrasonography, and magnetic resonance imaging (MRI). Modalities such as thoracic imaging techniques have proven to be valuable in diagnosing and predicting the outcomes of COVID-19. These imaging techniques are also effective in monitoring the health of COVID-19 survivors who experience long-term symptoms [6].

To train the CompLung structure, Pardyl, Adam et al. used the publicly available LIDC-IDRI dataset along with lung segmentation masks to improve the performance and interpretability of CompLung in comparison to prior techniques used to diagnose lung cancer [7]. Garstka et al. (2020) trained a self-constructed convolutional neural network on a relatively short dataset to classify lung X-ray images. They conducted a comparative analysis to evaluate the impact of data augmentation on the model’s performance and its ability to prevent overfitting. The categorization procedure achieved 85% accuracy with sensitivity of 0.95 [8]. Nurzynska et al. proposed a parameterized pipeline for the classification of whole slide images (WSI) in order to determine whether using many layers of imaging enhances the accuracy in classifying slides. The pipeline integrated a convolutional neural network (CNN) to categorize tiles within each image layer, leading to the creation of an AFB possibility score heatmap. They subsequently input the features generated from the heatmap into a whole slide image (WSI) classifier. The findings suggested that the process of acquiring a single layer can generate bias, also known as a sampling error, in the whole slide image (WSI). Either multilayer or extended-focus acquisition can reduce this bias [9]. Certain measures were employed to mitigate the spread of tuberculosis, a prevalent mycobacterial infection that impacts human health, typically using Ziehl–Neelsen (ZN)-stained slides to identify acid-fast mycobacteria (AFB) in tissue sections. These slides display vibrant red AFB against a blue background. Yang, Mu et al. suggested a machine learning pipeline that can correctly label digitized ZN-stained slides as either AFB-positive or AFB-negative. For tile recognition, the pipeline consists of two convolutional neural network (CNN) models. They assessed the work on a distinct set of tiles, yielding F1 ratings of 99.03% and 98.75%, respectively [10].

Artificial intelligence (AI) and machine learning (ML) have enabled significant advances in recent years, offering promising solutions to address the challenges associated with respiratory diseases. Among these advances, graph neural networks (GNNs) have emerged as a revolutionary system in medical image classification tasks. GNNs, originally designed for the analysis of graph-structured data, have emerged as a powerful type of network in medical imaging, where the relationships and connections within the data significantly improve the accuracy of diagnosis [11]. GCNs, or graph convolutional networks, go beyond traditional graph embedding techniques like Deepwalk in that they focus on creating a low-dimensional network representation while excluding node features [12,13]. Graph representative learning techniques are proven to represent comparable or superior classification performance for various medical tasks, including the signal and image domains [14,15,16].

Automatic diagnosis methods often use deep learning algorithms instead of reverse transcription polymerase chain reaction (RT-PCR) to diagnose chest X-ray images [17]. Chest scans are effective in identifying COVID-19 through visual markers such as the ground-glass opacity or hazy dark patches on the lungs, helping to distinguish affected individuals from healthy control individuals [18]. In this context, automated diagnosis systems frequently use chest computed tomography (CT) scans because of their high sensitivity and speed [19,20].

In contrast to traditional classification models, convolutional neural networks (CNNs) can work in the original high-dimensional space because they can be abstract. This implies that the preprocessing of the input image is not necessary [21,22].

Yang et al. [23] investigated and compared several deep learning-based methods using X-ray and CT scan images in the medical field to detect COVID-19 infection. The researchers employed four powerful pre-trained CNN models, DenseNet121, VGG16, ResNet50, and ResNet152, for the binary classification of COVID-19 from CT scan images. The F1-score and accuracy achieved were over 96%, indicating that the listed transfer learning techniques supported tasks with relatively limited data and reduced the training time.

Alrahhal and Supreethi [24] have developed a diagnostic system designed for the identification of COVID-19 based on CT scan images. The ResNet50 architecture improves feature extraction by producing a graphical representation of oriented gradient descriptors based on the visual phrases’ content. Subsequently, they employed an adaptive boosting classification model to identify the existence of COVID-19. They implemented this methodology on CT scans and an X-ray database, and the results were better when compared to other similar methods.

Xu et al. [25] preprocessed CT scan images to segment them into several image cubes using a 3D-CNN model. Then, using the extracted features from the ResNet18 model, a Bayesian learner categorized all of the image patches as COVID-19, influenza, and normal. They described their database, which featured 175 images in the normal category, 224 images in the viral pneumonia category, and 219 images in the COVID-19 category.

Yang et al. [26] used data augmentation and transfer learning to address the issue of overfitting that can easily emerge from the training of deep learning (DL) models on a limited dataset. They trained a DenseNet model on sample images from chest X-ray scans and then improved the pre-trained network on the COVID-19 dataset. Their supplied dataset featured 195 images in the normal category and 275 images in the COVID-19 category.

Song et al.’s method [27] offered three primary steps. In the first step, they extracted the major lung areas, and then they executed the segmentation process with the lungs themselves. To obtain the top-K data from the CT scans and provide image-level predictions, in the second step, they created a Detail Relation Extraction Neural Network (DRE-Net). Third, they combined the image-level predictions to achieve patient-level diagnoses. Their database consisted of 86 images for the normal group, 101 images for viral pneumonia, and 88 images for the COVID-19 category.

Zheng et al. [28] proposed a DeCoVNet with three steps. The first step was the network branch, and the second step comprised two 3D residual blocks (ResBlocks). The third step employed a progressive classifier (ProClf), utilizing a softmax activation function in the fully connected layer and 3D convolution layers. Their CT scan database consisted of 229 and 313 images for the normal and COVID-19 categories, respectively. Li et al. proposed COVNet, a method based on the RestNet-50 architecture [29]. This model generates features from the required CT slices. They then employed a max-pooling strategy to combine the features obtained from each individual slice. They utilized the softmax activation and a fully connected layer to provide a probability score based on the finalized feature map. Their dataset contained 1325, 1296, and 1736 images categorized as normal, COVID-19, and pneumonia, respectively.

Ucar et al. have suggested a deep learning method with a segmentation-based approach for COVID-19 disease detection based on CT scans [30]. To construct the suggested model, they changed the encoder part of the segmentation from the U-Net model. They used the deep learning models ResNet101, VGG16, DenseNet121, EfficientNetB5, and InceptionV3 in the encoder section. Then, using the majority voting rule, they combined the results of each updated U-Net model to determine a final conclusion. The experimental results showed that the suggested model had 99.38% specificity, 89.13% sensitivity, and an 85.03% segmentation Dice score.

A variety of studies aiming to detect lung disease, especially COVID-19 cases, use graph-induced approaches, such as graph convolutional networks (GCNs) [31]. Specifically applicable to 3D-CT images, a 3D-CNN architecture extracts visual features that enable the construction of COVID-19 graphs, designated as inputs to GCNs. However, due to its high computational complexity, the 3D-CNN is found to be less accurate, use significantly more memory, and require large datasets.

In the current study, we propose a COVID-19 diagnosis framework that combines the U-Net architecture with a feature-extracted GCN. With this, we attempted to reduce the high level of complexity in the data and explore higher-order patterns, which would improve the classification performance. By merging a graph convolution network with a U-Net model that is input with CT scan images, we aimed to extract pixel-level connectivity information based on their neighboring patterns to improve the predictions for the detection and classification of lung diseases. The graph-induced approach gives promising results and is applicable to imagery tasks related to medical or spectral images in future works.

## 2. Data and Methods

### 2.1. Dataset

**Figure 1:**
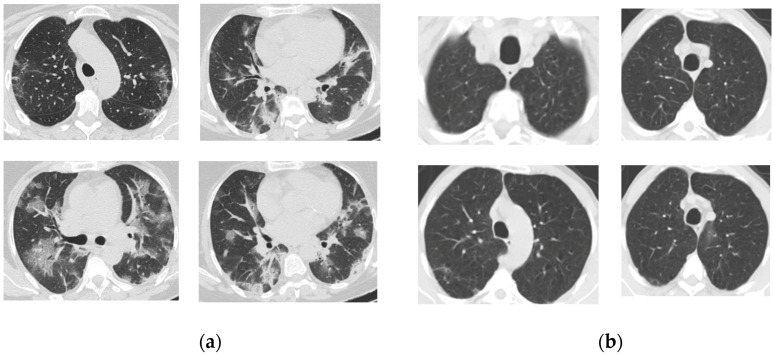
(**a**) COVID-19 and (**b**) non-COVID-19 samples [32].

We validate the proposed approach by employing a publicly accessible repository of SARS-CoV-2 CT scan images. The dataset, available on Kaggle, has a total of 2482 CT scans, each taken from a single patient, containing 1252 CT scans that are positive for SARS-CoV-2 infection and 1230 CT scans for patients not affected by SARS-CoV-2. The whole dataset is reported to be collected from real patients in hospitals from Sao Paulo, Brazil [32,33,34]. Figure 1 exhibits a selection of CT scan images depicting both COVID-19 and non-COVID-19 instances.

### 2.2. Overview

**Figure 2:**
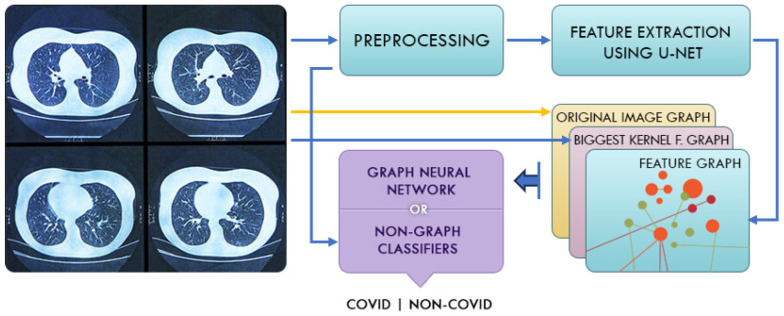
Block diagram of the proposed method.

We briefly explain the current study’s methodology as follows. Initially, we run a preprocessing procedure for each input CT scan image, which includes filtering, normalization, and data augmentation, to enhance the abstraction capacity of the 3D-CT image. Next, we detect images using nuclei and train the U-Net model on the dataset. The U-Net model performs the feature extraction and segmentation of the input CT images, yielding a graph-based feature map for each patient. We classify the 3D-CT images for diagnosis by feeding the modified GCN, known as the “feature-extracted GCN” (FGCN), with the graph-structured input. Figure 2 presents the block diagram of the proposed method.

### 2.3. Preprocessing

The preprocessing steps aim to enhance the abstraction of the 3D-CT images obtained from patients. Preprocessing includes three phases, listed as follows.

- Filtering: This process enhances or improves the quality of the 3D-CT scan images. Therefore, during filtering, a set of rules determines the value of any given pixel within the output image by calculating the values of the pixels in its vicinity.
- Normalization: This process modifies the range of pixel intensity levels. We also refer to it as “contrast stretching” because it enhances the image’s contrast.
- Data Augmentation: This is considered the most commonly used preprocessing technique for the U-Net model, aiming to improve the size of the training dataset by applying many modifications, e.g., rotation, translation, several types of symmetry, etc. Accordingly, it reduces the training’s sensitivity to noise and overfitting compared to the original input.

### 2.4. Feature Extraction Using the U-Net Model

**Figure 3:**
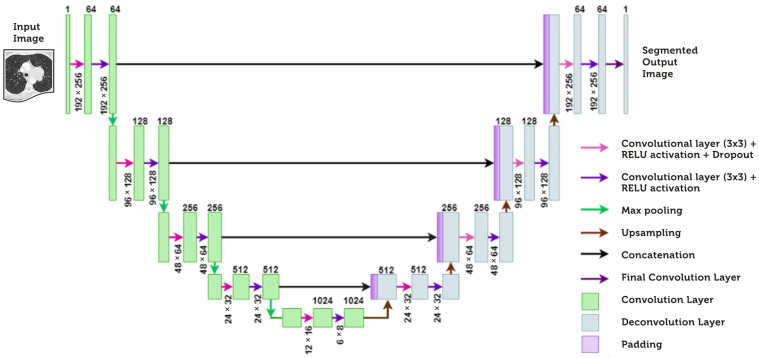
Feature extraction in U-Net architecture.

Feature extraction strategies are critical in developing capabilities that will be useful in classifying and popularizing images. Using the U-Net model, we extract features from 3D-CT scan images. For biological image segmentation, U-Net primarily uses a straightforward structure with high flexibility and excellent pixel-level segmentation results. We intentionally chose the symmetrical architecture of U-Net to assist in image processing and computer vision tasks that require feature extraction and localization, context modeling, and precise resolution recovery. It leverages the benefits of both the encoder and decoder pathways to produce accurate and detailed output predictions. This model includes an encoder part (also called a contracting path; left side in Figure 3) and a decoder part (also called an expanding path; right side in Figure 3). Each of the boxes in the U-Net structure is a multi-channel feature map. The arrows represent various operations, such as max pooling, convolution, and copy and crop [35].

### 2.5. Classification Using FGCN

#### 2.5.1. Graph Conversion

**Figure 4:**
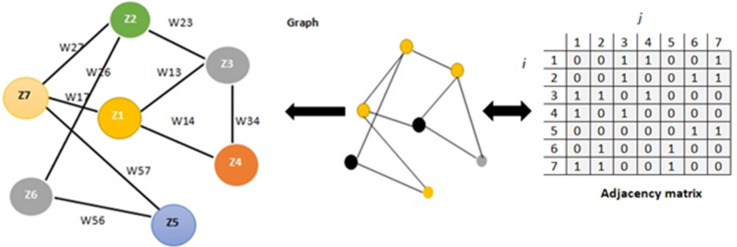
Directed graph example and its accompanying adjacency matrix modified from an image.

The formula $G=\left(N\;,\;E\;,\;A\right)$ represents a graph formula, with *N* denoting the collection of nodes, (*E*) is the edges between nodes, and (*A*) is the adjacency matrix. The adjacency matrix $A_{ij}$ represents the connections between each node pair, with an entry indicating the weight of the link between *i* and the *j*-th node, which is the intersection of the *i*-th row and the *j*-th column. We can construct the graph using the Pearson correlation, the k-nearest neighbor (KNN) algorithm, or distance-based approaches to calculate the entries [36]. Figure 4 represents a sample of a graph with six nodes and the edges bridging them. We also provide the matrix that represents the adjacency of the graph nodes.

The convolution process for spectral-based GCNs is constructed with the Fourier transform by calculating the graph Laplacian’s eigen-decomposition [37]. The description of graph Laplacian normalization is L=(IN−D−12 A D−12=U∧UT), where D is the degree matrix and *A* is the adjacency matrix. The columns of U constitute the eigenvector matrix and Ʌ is the diagonal matrix, which contains its eigenvalues. This operation is described as the multiplication of a signal $\left(X\in \;R^{N}\right)$, and the scalar for every node with a filter $\left(g_{\theta }=diag\left(\theta \right)\right)$ is parameterized by $\left(\theta \in R^{N}\right)$.

(1) $$g_{\theta }*x=Ug_{\theta }\left(\wedge \right)U^{T}x$$

Defferrard et al. proposed ChebNet, which avoids computing the Fourier basis by approximating the spectrum filters by shortened Chebyshev polynomials. The procedure is defined as using a Chebyshev polynomial $T_{m}(x)$ of order m [38].

(2) $$g_{\theta }*x\approx \;\sum_{m=0}^{M-1}\theta_{m}T_{m}{\left(\bar{L}\right)}_{x}$$

In Equation (2), $\bar{L}$ is the diagonal matrix of a scaled eigenvalue with a formula $\bar{L}=2L∕\lambda max-I_{N}$. The largest eigenvalue of *L* is indicated by the symbol max. The Chebyshev polynomials are denoted by $T_{m}\left(x\right)=2xT_{k-1}\left(x\right)-T_{k-2}(x)$, where $T_{0}\left(x\right)=1$ and $T_{1}\left(x\right)=x$.

ChebNet uses Chebyshev polynomials, eliminating the need to compute the Laplacian matrix’s eigenvectors and reducing the computational cost. A graph pooling layer in the GCN decreases the size of the graph and increases the receptive field of the graph filters. The graph convolutional network’s preceding layer combines the feature vectors to create a singular vector, which then feeds into a fully connected layer to produce the classification results.

#### 2.5.2. Graph Convolutional Network (GCN)

**Figure 5:**
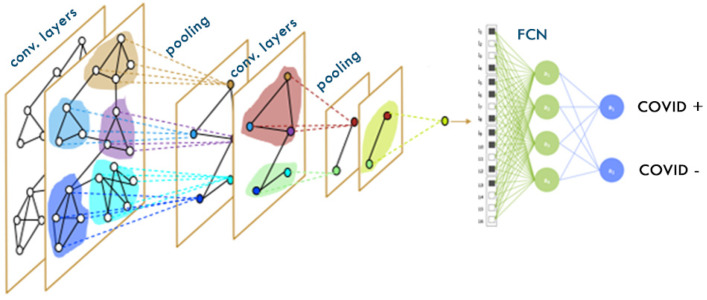
Evaluation of graph input data with a graph convolutional network.

A spectral GNN with mean pooling accumulation is referred to as a GCN. Kipf and Welling [39] proposed the GCN using a limited first-order estimate of the spectral convolution layers on graphs. Figure 5 illustrates a straightforward layer-wise propagation algorithm that converts the connections between the nodes in the graph design into node features. By assuming a λ≈2 reduction in the size of the convolutional filter to K = 1, we can simplify Equation (2) and address the issue of overfitting in graphs with a large degree distribution of nodes in their local neighborhood structure [39].

(3) gθ∗x≈θ 0+θ1L−INx=θ0x+θ1D−12AD−12x

The values of $\theta_{0}$ and $\theta_{1}$ are not limited in Equation (3). The GCN additionally assumes that $\theta =\theta_{0}-\theta_{1}$ so as to restrict the number of parameters and prevent overfitting. This results in the formulation of a graph convolution as follows:

(4) gθ∗x≈θIN+D−12  A  D−12x

This process will result in numerical instability and the explosion or disappearance of the gradients if it is stacked.

In order to apply the signal definition, we require $x\in R^{F*C}$ with C input channels and F filters for feature extraction. Kipf and Welling [39] expand this formula as follows:

(5) Z=D−12AD−12  xθ

where Θ ∈ R^N*F^ is the signal matrix formed via convolution and Θ ∈ R^CXF^ is the matrix formed by filterbank settings.

The classification task is performed using the modified version of the GCN, labeled the feature-extracted GCN (FGCN) in the current study. The steps involved in the FGCN are given below.

Step 1: The pyramid features are extracted from the CT scan images using a U-Net model at different layers and with different sizes of kernels.
Step 2: These extracted features are combined to form an adjacency matrix.
Step 3: The COVID-19 CT scan images are converted into a graph, which is formed using a combined adjacency matrix representing the edges and extracted features.
Step 4: The original CT scan graphs and the largest kernel graph are formed.
Step 5: These three graphs are combined to form one block of graph input, which is passed through the GCN with an extra dropout layer to reduce overfitting.
Step 6: Finally, image classification is performed to detect whether the patient is affected by COVID-19.

### 2.6. Baseline Learning Models

Deep learning has employed several CNN models to tackle significant tasks in image processing, like medical image classification and object detection. Medical image classification tasks employ an extensive variety of customized CNN models, and we also examined some CNN-based deep learning models on the same dataset with the same FGCN model; we provide a brief explanation below.

DenseNet201: This is a convolutional neural network (CNN) model that has been specifically designed with 201 layers. We utilized a previous version of the model that experienced training on a dataset including more than one million images taken from the ImageNet database. The pre-trained network employs a feed-forward connection between the layers and possesses the capability to categorize images into 1000 different classes. This includes the identification of various items, as well as the diagnosis of diseases in medical image classification [40].

VGG16: This is a widely used transfer learning model that consists of 13 convolutional layers and three fully connected layers. Each convolutional layer has 3 × 3 kernels, while the pooling layers have 2 × 2 parameters. Blocks 1 to 5 consist of many convolutional layers and one pooling layer, which together form the convolutional and pooling layers of VGG16. Block 1′s two convolutional layers use 16 kernels to extract features, while the pooling layer reduces the image size. The design of subsequent blocks is uniform, except for the fact that blocks 1 and 2 utilize two convolutional layers, while blocks 3–5 utilize three convolutional layers, each with a distinct kernel size. This serves to deepen the network and boost its accuracy. Three fully interconnected layers are combined in the last stage to provide features classified into two distinct classes [41].

InceptionV3: This is a convolutional neural network model that has 48 layers and can learn to detect difficult patterns as well as features in medical images. This model’s capacity to adjust a huge amount of data and manage images of different shapes and qualities is one of its primary strengths. This is critical in the field of medical image processing because of the wide range of image sizes, quality levels, and resolutions. This model typically has three convolutions of varying shapes and a maximum of one pooling layer [42].

ResNet50: This convolutional neural network model specifically tackles the problems of vanishing or expanding gradients. ResNet50 presents the idea of a residual neural network, which is a convolutional neural network consisting of 50 layers that emphasizes the acquisition of residuals rather than features [42].

NASNet Mobile: Google introduced NASNet Mobile, a powerful and well-designed solution that utilizes extensive processing capabilities. It treats the task of finding the optimal structure for a convolutional neural network (CNN) as a problem to solve through reinforcement learning. The primary objective of this model is to determine the most efficient configuration for each parameter in the search space, such as the filter sizes, output channels, strides, and number of layers. We designed every search operation in the reinforcement learning framework to evaluate and reward the correctness of this model on the given dataset.

NASNet achieved a cutting-edge outcome in the ImageNet competition. Nevertheless, the model’s extensive computational requirements restrict its applicability to a limited number of classification tasks, while simultaneously enhancing the efficiency of the model’s architecture [43].

EfficientNet: This is a convolutional neural network (CNN) approach that uses a compound coefficient to appropriately scale the dimensions of depth, breadth, and resolution. EfficientNet’s scaling technique uniformly scales the depth, width, and resolution of the network by using a predefined set of scaling coefficients. This is different from the conventional approach, where these variables are scaled without a specific pattern. EfficientNet employs a compound coefficient to consistently scale all dimensions and resolutions of the network. The mixed scaling approach operates on the principle that larger input images require an increase in the number of layers to expand the network’s receptive field and a greater number of channels to capture more intricate shapes within the entire image [44].

### 2.7. Experimental Environment

The experimental setup employs the Python environment, Torch package, SciPy, and NumPy to train the proposed deep learning models. The FGCNs were executed on a standard system, which included 64 GB of RAM, an Intel Core i7-12700HQ CPU operating at a frequency of 2.8 GHz, and an RTX 3080 GPU with 8 GB of memory.

### 2.8. Hyperparameters

The recommended specifications for the GCN structure include 50 epochs each with 64 neurons per layer. We set the learning rate at 0.0001 and used a dropout rate of 0.1. Concretely, we resized each image to a fixed resolution of 512 × 512 pixels. To mitigate overfitting in all pre-trained models using a particular dataset, we employed a random 80–20 split, allocating 80% for training and 20% for testing. The software environment for the execution of the Python 3.11 code included Spyder and Jupyter Notebook, both of which are part of the Anaconda distribution. 

We used the Rectified Linear Unit (ReLU) [45] with the Adam optimizer [46] to activate each of the convolutional layers in the transfer learning models examined in this study. In addition, we incorporated a dropout layer with a rate of 0.3, which randomly deactivated thirty percent of the neurons throughout each training session. This mitigated the risk of overfitting on the training dataset.

## 3. Results and Discussion

This section shows the success of our suggested method (FGCN) in classification using different general DL models, such as VGG16, InceptionV3, NasNet Mobile, ResNet50, and DenseNet201. Table 1 displays several metrics, including the sensitivity, precision, specificity, F1-score, and accuracy, used to evaluate the performance of each model.

Based on the findings, the suggested model (FGCN) demonstrates classification accuracy of 99.19%, outperforming the other examined models. The models that achieved the highest levels of success were VGG16, ResNet50, and DenseNet201, with accuracy of 97.38%, 96.37%, and 96.18%, respectively. InceptionV3 and EfficientNetB0 were unable to compete with the other models because their accuracy values were below 92%. The NASNet Mobile model had the lowest capabilities, with scores of approximately 80%. The suggested model exhibits exceptional sensitivity, precision, specificity, and F1-score metrics, all over 99%, as demonstrated.

**Figure 6:**
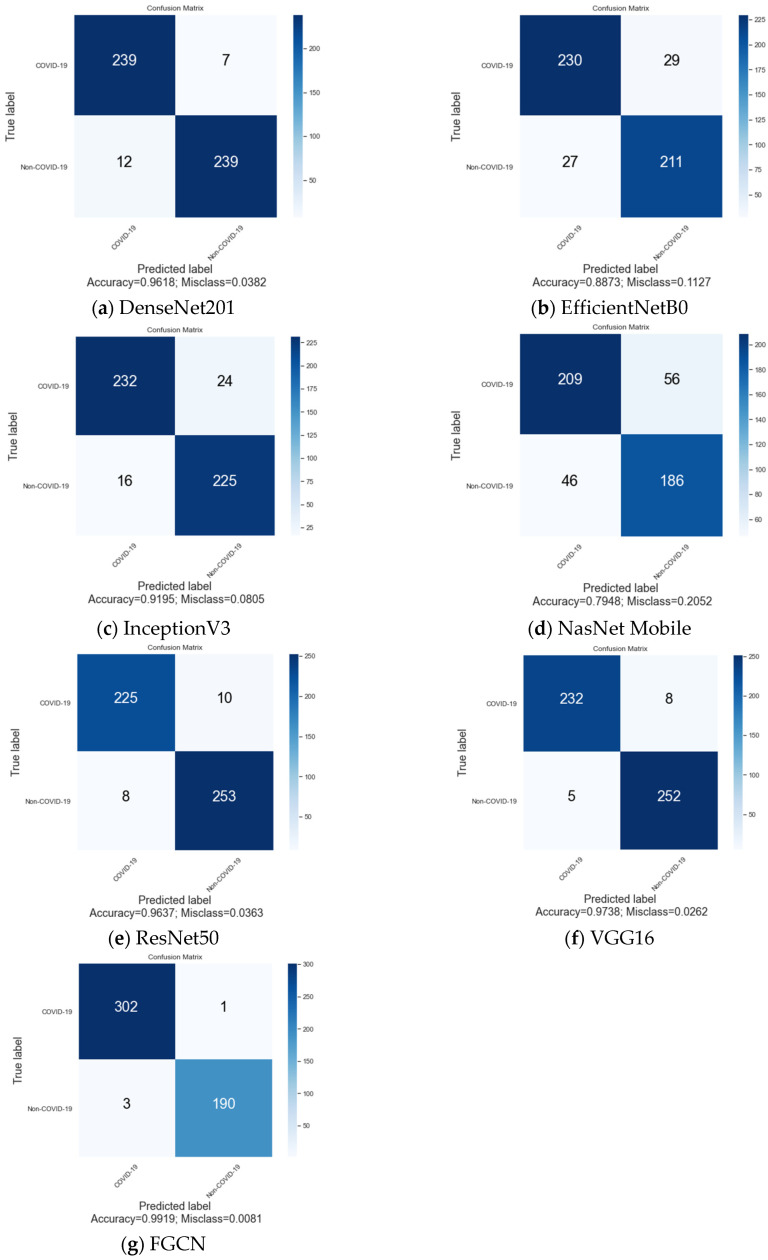
Confusion matrices for the models tested.

Figure 6 provides a detailed overview of the classification metrics mentioned in Table 1 by displaying the confusion matrices for all of the examined models. The proposed FGCN model incorrectly classified only one and four cases into the positive and negative classes, respectively. The models that had the most similar performance were DenseNet, which misclassified seven positive cases, and VGG16, which misclassified five negative cases. The models with the lowest accuracy demonstrated a balanced distribution of misclassifications between the two classes.

Table 2 provides a brief description of the most recent deep learning algorithms used to detect COVID-19 through the analysis of computerized X-ray (CXR) or CT images. Compared to previous research, the proposed FGCN model provides similarly excellent performance, characterized by its unique feature of integrating the U-Net architecture with a GCN. Furthermore, it demonstrates the efficacy of a graph-based method for the diagnosis and identification of complex patterns associated with lung diseases.

## 4. Conclusions

This paper introduces a novel approach to COVID-19 detection by combining the U-Net model with a GCN to create a feature-extracted GCN (FGCN). We employ the U-Net model for both image segmentation and feature extraction. We use the generated features to create an adjacency matrix holding the graph structure. The GCN also receives the original image graph and the largest kernel graph. To establish unified input graph data, we combine these graphs and pass them through a graph convolutional network (GCN) along with a dropout layer. This helps to reduce overfitting while diagnosing COVID-19. The resulting model diverges from the existing works as being the first approach to evaluate CT images of lungs structured as a graph representation of features, classified by a graph neural network model, also outperforming the most recent models proposed for COVID-19 detection in the literature.

Moreover, the proposed FGCN model is compared with six commonly used transfer learning models, namely DenseNet201, EfficientNetB0, InceptionV3, NasNet Mobile, ResNet50, and VGG16. The FGCN is found to overperform these transfer learning models. These outcomes underscore the abstraction potential of the graph-induced technique, making it suitable for similar medical diagnosis tasks.

## Figures and Tables

**Table 1:** Classification results achieved for SARS-CoV-2 CT dataset using several pre-trained deep learning models.

DL Model	Sensitivity	Precision	Specificity	F1-Score	Accuracy
DenseNet201	0.9715	0.9522	0.9522	0.9618	0.9618
EfficientNetB0	0.8880	0.8949	0.8866	0.8914	0.8873
InceptionV3	0.9062	0.9355	0.9336	0.9206	0.9195
NasNet Mobile	0.7887	0.8196	0.8017	0.8039	0.7948
ResNet50	0.9574	0.9657	0.9693	0.9615	0.9637
VGG16	0.9667	0.9789	0.9805	0.9728	0.9738
FGCN	0.9967	0.9905	0.9845	0.9936	0.9919

**Table 2:** Comparison some of DL models based on performance on various image datasets for COVID-19 detection.

Reference	Dataset	Model	Accuracy	Sensitivity	Precision	Specificity	F1-Score	AUC
[47]	CT scans (4382 COVID-19, and 9369 non-COVID-19)	U-Net++	95.2	100	-	93.6	-	
[48]	Total of 1918 CT scans	DCN	95.99	89.14	-	98.04	-	97.55
[49]	CT scans (449 COVID-19 and 595 non-COVID-19)	Two U-Nets	86	94	-	79	-	93
[50]	COVID-19 CT scans	U-Net+	82.9	97.4	-	92.2	-	-
[51]	CT scans (521 COVID-19 and 665 non-COVID-19)	EfficientNetB4	87	89	-	87	-	90
[52]	CT scans (1262 COVID-19 and 1230 non-COVID-19)	DenseNet201	96.25	96.29	96.29	96.21	96.29	-
[53]	CT scans (108 COVID-19 and 912 non-COVID-19)	Xception	99.02	96.29	96.29	96.21	96.29	-
[29]	CT scans (468 COVID-19 and 2996 non-COVID-19)	ResNet50	89.5	87	-	92	-	95
[50]	CT scans (723 COVID-19 and 413 non-COVID-19)	U-Net and ResNet50	94.8	97.4	-	92.2	-	-
[54]	CT scans (360 COVID-19 and 34 non-COVID-19)	VGG16	91	94	100	-	97	-
[27]	CT scans (219 COVID-19 and 399 non-COVID-19)	ResNet50	86	96	79	-	-	9596
[55]	Consists of 1065 CT scans	CNN	83	84	-	80.5	-	-
[56]	CT scans (1493 COVID-19 and 4594 non-COVID-19)	Inception-ResNetV2	92.18	92.11	92.38	96.06	-	-
[57]	CT scans (260 COVID-19 and 600 non-COVID-19)	VGG19	89.3	89	90	-	90	-
[58]	CT scans (1252 COVID-19 and 1230 non-COVID-19)	DarkNet19 with repeated holdout 10FCV	98.91	98.96	-	98.86	0.99	-
[59]	CT scans (313 COVID-19 and 229 non-COVID-19)	U-Net and CNN	90.9	90.7	-	91.1	-	95.9
[60]	CT scans (192 COVID-19 and 145 non-COVID-19)	nCOVnet	97.62	97.62	-	78.57	-	-
[61]	CT scans (347 COVID-19 and 397 non-COVID-19)	ResNet18	78.29	76.9	81	79.9	78.9	83.82
**Current study**	**CT scans (1252 COVID-19 and 1230 non-COVID-19)**	**FGCN**	**99.19**	**99.67**	**99.05**	**98.45**	**99.36**	

## Data Availability

The original contributions presented in the study are included in the article, further inquiries can be directed to the corresponding author.
